# Investigating the development of causal inference by studying variability in 2- to 5-year-olds' behavior

**DOI:** 10.1371/journal.pone.0195019

**Published:** 2018-04-02

**Authors:** Tessa J. P. van Schijndel, Kim Huijpen, Ingmar Visser, Maartje E. J. Raijmakers

**Affiliations:** 1 Research Institute of Child Development and Education, University of Amsterdam, Amsterdam, The Netherlands; 2 Leiden University Graduate School of Teaching (ICLON), Leiden University, Leiden, The Netherlands; 3 Research Priority Area Yield, University of Amsterdam, Amsterdam, The Netherlands; 4 Developmental Psychology, University of Amsterdam, Amsterdam, The Netherlands; 5 Amsterdam Brain and Cognition, University of Amsterdam, Amsterdam, The Netherlands; 6 Education Sciences, Leiden University, Leiden, The Netherlands; TNO, NETHERLANDS

## Abstract

This study investigated the development of young children’s causal inference by studying variability in behavior. Two possible sources of variability, strategy use and accuracy in strategy execution, were discriminated and related to age. To this end, a relatively wide range of causal inference trials was administered to children of a relatively broad age range: 2- to 5-year-olds. Subsequently, individuals’ response patterns over trials were analyzed with a latent variable technique. The results showed that variability in children’s behavior could largely be explained by strategy use. Three different strategies were distinguished, and tentative interpretations suggest these could possibly be labeled as “rational”, “associative”, and “uncertainty avoidance” strategies. The strategies were found to be related to age, and this age-related strategy use better explained the variability in children’s behavior than age-related increase in accuracy of executing a single strategy. This study can be considered a first step in introducing a new, fruitful approach for investigating the development of causal inference.

## Introduction

The question that has dominated the literature on children’s causal reasoning for the last decade is how new causal relations are learned and represented [1, 2]. A line of studies has investigated children’s ability to make causal inferences from a very young age on [3, 4, 5, 6, 7, 8, 9]. The majority of these studies have used the blicket detector paradigm to create an artificial, novel setting for testing this ability [4, 6].The paradigm consists of a machine that activates (lights up and plays music) when some objects (blickets), but not others, are placed on it. Each blicket detector trial measures a learner’s ability to make a specific type of causal inference, such as *screening-off*, *indirect screening-off*, or *backwards blocking*.

Even though this line of work did not focus on investigating the development of causal inference, it has uncovered some developmental differences. It has been shown that the majority of 19-, 24-, 30-month-olds [5, 8] and 3- to 5-year-olds [5, 7] are capable of making *screening-off* inferences. This implies *not* considering a target object to be a blicket in the context where the target object activates the machine in the presence of an established blicket, but not by itself [10]. It has been shown that in contrast to the majority of 19-month-olds [8], the majority of 24-month-olds [8], and 3- and 4-year-olds [9] are capable of making *indirect screening-off* inferences. This implies considering a target object to be a blicket in the context where the target object activates the machine in the presence of a second object, and the second object does not to activate the machine by itself. Additionally, it has been shown that in contrast to the majority of 3-year-olds, the majority of 4-year-olds are capable of making *backwards blocking* inferences [9]. This implies *not* considering a target object to be a blicket in the context where the target object activates the machine in the presence of a second object, and the second object activates the machine by itself. These findings have led researchers to stress the need for future research to further investigate the developmental trajectory of causal inference, e.g. [9].

In previous work on preschoolers’ causal inference, conclusions on the existence of developmental differences have been based on the average behavior of age groups. This approach stands in contrast with the sizable variability in behavior within age groups that has been observed in these studies. Within groups that were considered to be capable of making a specific type of inference, percentages of succeeding children ranged from 72 to 100 for *screening-off* inferences [5, 7, 8] and from 76 to 100 for *indirect screening-off* and *backwards blocking* inferences [8, 9] As mapping the variability in children’s behavior is considered of great importance in studying development [1], the goal of the present study is to further investigate the development of causal inference by studying variability in behavior.

Variability in children’s behavior could originate from different sources. Possibly subgroups of children apply different strategies for solving causal inference problems (we will refer to this source of variability as *strategy use*). By “strategy” we mean a process, either conscious or unconscious, resulting in a certain approach to solving causal inference trials, cf. [11]. Different strategies either imply the use of different learning mechanisms, such as Bayesian methods or causal learning on the basis of associative models (for an overview see Gopnik et al., 2004) [3], or the use of different task-related strategies, such as imitation of the experimenter or guessing, e.g. [5, 8]. Alternatively, variability in behavior is explained by children making errors in applying strategies (we will refer to this source of variability as *accuracy in strategy execution*). Differences in accuracy can, for example, be related to differences in information processing abilities [9]. To render a complete picture of the development of children’s causal inference, it is important to discriminate between these sources of variability and relate them to age. From this analysis roughly two developmental models could be proposed: either children of different ages apply different strategies (*strategy use*), or children of different ages apply the same strategy, but become better at executing this strategy with age (*accuracy in strategy execution*). Investigating which of these models best describes development is an important first step in further investigating the development of children’s causal inference. Ultimately, one would want to know what strategies children apply, and how strategy use is related to age and the circumstances a child faces. However, before being able to study these interesting, but highly complex issues, a necessary first step is to determine whether variability in behavior can be explained by *strategy use* at all.

The approach that has been used in previous work on children’s causal inference, does not allow one to discriminate between variability in responses stemming from *strategy use* and variability stemming from *accuracy in strategy execution*, because average behavior was analyzed over no more than one or two causal inference trials [3, 4, 5, 6, 7, 8, 9]. Therefore, in the present study, we look at individuals’ response patterns over a larger number of trials. To this end, children of a relatively broad age range, 2- to 5-year-olds, are administered a relatively wide range of causal inference trials: a *screening-off* trial [5], two versions of an *indirect-screening-off* trial [9, 7], a *backwards blocking* trial [9], and a *non-causal association* trial [4, 12] (see Method). Mainly, these trials are selected for their frequent use and tendency to bring about variable responses in previous work (see Method). Individuals’ response patterns over trials are analyzed with a latent variable technique [13, 14] (see Method). The methodological approach allows us to map the variability in children’s causal inference behavior by comparing the relative fit of models that explain variability in responses in different ways, and this way to contribute uniquely to the research on the development of this ability. The present study’s first research question is: Can variability in 2- to 5-year-olds’ behavior be explained by *strategy use*? To answer this question, the fit to the data of a 1-class-model (not indicative of variability being explained by *strategy use*) will be compared to the fit of multiple-class-models (indicative of variability being (partially) explained by *strategy use*). The detection of different subgroups producing comparable responses for the four (different) trials is a first indication of *strategy use*. Stability of responses over multiple, equivalent items (e.g., indirect-screening-off trials) would provide a stronger indication. However, this young age group does not allow for administering more trials. The second research question is: Is children’s *strategy use* related to age? To answer this question, the fit to the data of models with and without age as a covariate on the class membership probabilities will be compared. The third question is: Does age-related *strategy use* better explain the variability in children’s behavior than an age-related increase in *accuracy of executing* a single strategy? To answer this question, the fit to the data of a 1-class-model (indicative of variability being explained by *accuracy of execution*) and a multiple-class-model (indicative of variability being explained by age-related *strategy use*) with age as a covariate on the response probabilities will be compared. Please see the Methods and Materials and Results sections for further explanation of the latent variable techniques, including the model comparisons. In the Discussion we will elaborate on the selected model and provide directions for future research by speculating on possible interpretations of the strategies of the model.

## Material and methods

### Participants

The final sample consisted of 90 children (40 boys and 50 girls): twenty-three 2-year-olds (*M* = 30.04 months, *SD* = 3.16), twenty-three 3-year-olds (*M* = 40.87 months, *SD* = 3.56), twenty-three 4-year-olds (*M* = 54.39 months, *SD* = 2.98) and twenty-one 5-year-olds (*M* = 67.33 months, *SD* = 2.44) who were recruited from three daycare centers and a primary school (in May and June 2007, and May and June 2012). Twenty-five other children were recruited, but excluded from the analyses due to experimental error (N = 5), missing values on test trials (N = 3), or failing the training trials (N = 17: fourteen 2-year-olds and three 3-year-olds; see Procedure).

For all participating children written consent from a parent or legal guardian was obtained. The study, including the consent procedures, was approved by the Ethics Committee of the Department of Psychology of the University of Amsterdam.

### Materials

The blicket detector [4, 6] was used. The detector was made of grey plastic with an orange top and measured 18.8 x 10.8 x 7 cm. A hand held remote control, hidden from the child’s view, was used to control whether objects placed on top of the detector activated it (it lit up and played music), or not. Objects that activated the detector did so as soon as they made contact with it, and deactivation immediately followed removal. Ten sets of unique objects (22 in total, 2–3 per set) were used: five sets of wooden blocks and five sets of plastic toys of different shapes and colors (two sets of fruits, one set of ducks and two sets of bowling pins). Each set was used for one trial (see below), the composition of sets of blocks over trials was counterbalanced across participants.

### Procedure

Children were tested individually in a private room at their daycare center or school. The experimenter first introduced the blicket detector to the child: “We are going to play with this machine. The machine goes on if some things are placed on top of it. If other things are placed on top of the machine it does not go on. You need to help me figure out which things make the machine go”.

Children were then administered ten trials in two training-test blocks: three training trials (A, B, C), two test trials (1, 2), two more training trials (D, E) and three more test trials (3, 4, 5). The training trials were used to familiarize participants with the two types of responses that were required for the different test trials: an intervention to make the machine go (test trial 1, 2 and 5) and a verbal response to the question “Does this one make the machine go?” (test trial 3 and 4). Additionally, the training trials served as control trials to ensure that children were on-task and understood the nature of the task. In order to be included in the analyses, participants had to answer a minimum of four out of five training trials correct. Test trials were selected on the basis of their frequent use and their tendency to bring about variable responses in previous work. A third criterion for selection was that trial types had previously been used to investigate the use of learning mechanisms or task-related strategies (see Discussion for more information on the complexity of this topic).

In trials A, B, C, 1 and 2 children were asked the question “What makes the machine go? Can you point to it?” before they were asked to make the machine go. This was done to check whether responses on the go-questions were influenced by children’s limited motor skills. If a child intended to respond to a go-question by putting multiple objects on the machine, but as a result of limited motor skills put the objects on the machine one by one, the experimenter could have slid the tray away after the first object and the child’s reasoning would not have been captured by the trial. The pointing-questions served as a check whether this was the case. However, as a considerable part of the younger participants in this study did not understand the pointing-questions, the responses on this question were not used for further analyses. Below we briefly describe all trials, for more information on the administration protocol or scoring forms please see http://dx.doi.org/10.17504/protocols.io.ntydepw

#### Training trials A, B and C

For trial A the experimenter placed object X on the machine, it activated and she said: “See, it makes the machine go.” She then placed object Y on the machine, it did not activate and she said: “See, it does not make the machine go.” Subsequently she slid the tray with the detector and two objects alongside it towards the child and asked: “Can you make the machine go?” The child was allowed to make one response after which she slid the tray back to her side of the table. If the child placed the causally efficacious object (A) on the machine, it activated. Training trial B and C were similar to trial A, except that in trial B the second object was the causally efficacious one and in trial C the experimenter did not state anymore that objects did or did not make the machine go.

#### Test trials 1 and 2

Trial 1 was a *screening-off* trial [5]. The experimenter placed object X on the machine and it activated. She placed object Y on the machine and it did not activate. She then placed both objects on the machine together and it activated. This action was repeated. Subsequently she followed the procedure of training trial A and asked the child to make the machine go. On this trial, succeeding in making a *screening-off* inference would imply putting object X on the machine. Trial 2 was a variant of Schulz and Gopnik’s (2004) [7] *indirect screening-off trial in which children were not shown information about single candidate causes* (we will refer to this trial as the *indirect screening-off trial A* in the remainder of the paper). The experimenter placed object X and Z on the machine together and it activated. She placed object Y and Z on the machine together and it activated. She then placed object X and Y on the machine together and it did not activate. Subsequently she followed the procedure of training trial A and asked the child to make the machine go. On this trial, succeeding in making an *indirect screening-off* inference would imply putting object Z on the machine.

#### Training trials D and E

For trial D the experimenter placed each object on the machine by itself. Object X did not activate the machine, object Y did. Subsequently she showed the child object X and asked “Does this one make the machine go?” She then showed the child object Y and asked “Does this one make the machine go?” Trial E was similar to trial D, except that in this trial the first object was the causally efficacious one.

#### Test trials 3 and 4

Trial 3 was an *indirect screening-off* trial [9] (we will refer to this trial as the *indirect screening-off trial B* in the remainder of the paper). The experimenter placed two objects X and Y on the machine together and it activated. This was demonstrated twice. She then placed object X on the machine by itself and it did not activate. Subsequently she used the procedure of training trial D and asked the child if each object made the machine go. On this trial, succeeding in making an *indirect screening-off* inference would imply labeling object Y as a blicket. Trial 4 was a *backwards blocking* trial [9]. It was similar to trial 3, except this time when object X was placed on the machine by itself, it did activate. On this trial, succeeding in making a *backwards blocking* inference would imply not labeling object Y as a blicket.

#### Test trial 5

Trial 5 was a variant of Gopnik and Sobel’s (2000) [4] and Nazzi and Gopnik’s (2003) [12] *non-causal association* trials. The experimenter held object X slightly above the machine, pressed the top of the machine with her finger, and the machine activated. She repeated this procedure for object Y. She then held object Z slightly above the machine, placed her finger near the machine but did not press it, and the machine did not activate. Subsequently she followed the procedure of training trial A and asked the child to make the machine go. On the basis of the demonstration, object X and Y could be considered temporally associated objects and object Z a non-associated object. However, as the activation of the machine appeared to be caused by the experimenter’s action [12], succeeding in making a causal inference on this trial would imply using a finger press.

As in previous blicket detector studies no order effects were found, e.g. [8, 9], a fixed order of administration was used. This choice ensured that training trials in which a response type (intervention versus verbal response) was introduced preceded test trials in which this response type was required, and that test trial 5 with the finger press was always administered last, to avoid carry-over effects to other trials. The side of the detector where the causally efficacious object was placed over trials was counterbalanced across participants.

### Statistical approach

To investigate the variability in young children’s causal inference behavior, we used latent class analysis (LCA) [13, 14]. In previous research in the field of developmental psychology this approach has been used to investigate the variability in children’s (naïve) theories on several science subjects [15, 16, 17, 18, 19, 20, 21], and children’s transitive reasoning [22, 23], discrimination learning [24], and free classification [25]. This methodological approach has several advantages over using pattern matching techniques in which predefined strategies are matched to response patterns [26]. For example, it allows for the detection of anticipated as well as unanticipated strategies, it does not require the researcher to set an arbitrary criterion for correspondence between observed and expected responses to classify children in strategy groups, and it uses model selection techniques that allow for an optimal decision between goodness-of-fit and parsimony of the model (for an elaborate discussion see Van der Maas and Straatemeier, 2008) [27].

In the present study, LCA was used to describe children’s binary response patterns on the test trials in terms of latent classes, that is, strategies for causal inference. Latent class models (LCM) were fitted to the data by calculating Log Likelihood estimates of the model parameters with the package depmixS4 [28] for the R statistical programming environment [29]. LCM are defined by the number of latent classes (in the present study: 1–4 classes), and different types of parameters: class membership probabilities, response probabilities, and possible covariates on those probabilities. Class membership probabilities define the class sizes. Response probabilities of a class indicate probabilities of succeeding on specific trials given membership of that class, and can therefore be interpreted as accuracy parameters. Succeeding is defined as making the intended causal inference, such as screening-off or backwards blocking. In the Materials section the specific responses per trial consistent with making the intended causal inference are specified. When comparing nested models (in the present study: models with the same number of classes, but with or without covariates on the parameters), the optimal LCM is determined by a likelihood ratio test [30] (p. 141). A significant test implies a better fit of the more extended model (in the present study: the model with covariates). When comparing non-nested models (models with a different numbers of classes), the optimal LCM is determined by using the Bayesian Information Criterion (BIC) [31]: the LCM with the lowest BIC is considered to be the most parsimonious, best fitting model. We also calculated the Akaike’s Information Criterion, but because the model selection based on this criterion rendered the same results as the model selection based on the BIC criterion, we do not report the AIC’s. After model selection, strategy use was related to age by calculating the most likely class membership for each participant separately.

## Results

### General

To enable comparison of responses over the different test trials, we rescored children’s responses in a binary way, as succeeding or not succeeding. Succeeding was defined as making the intended causal inference, such as *screening-off* or *backwards blocking*, not succeeding included all other responses (see Method). Rescoring revealed a lack of variability in children’s responses on test trial 5, the *non-causal association* trial: only two children (2%) succeeded (61% used (an) associated object(s) or combined these with the finger, 33% used the non-associated object or combined this with (an) associated object(s)/ the finger, 3% gave a response that could not be scored). In contrast, Nazzi and Gopnik’s (2003) [12] results showed more variability in responses on the *non-causal association* trial (27% of the children succeeded). These differences can be explained by differences in the procedures of both studies, such as by the number of times the trial was administered and the number of objects children could choose from to make the machine go. Because of the lack of variability in children’s responses on the present study’s *non-causal association* trial, this trial was excluded from further analyses. Last, we checked whether the compositions of sets of blocks over the trials or the side of the detector where the causally efficacious object was placed had affected the proportions of children succeeding on the four remaining test trials. This was not the case.

### Comparison results to previous studies

Several test trials (trial 1: *screening-off*, trial 3: *indirect screening-off B*, and trial 4: *backwards blocking*) had been administered with the exact same procedures as in previous studies. Hence, before performing the individual difference analyses, we compared the proportions of children succeeding on these trials in the present study to those of children of comparable ages in previous studies (see Table 1). The proportion of 2-year-olds making a *screening-off* inference did not differ between this study and Sobel and Kirkham’s (2006) study [8]. The proportions of 3-year-olds making *indirect screening-off* and *backwards blocking* inferences did not differ between this study and Sobel et al.’s (2004) study [9]. The proportion 4-year-olds making an *indirect screening-off* inference did not differ between this study and Sobel et al.’s study. The proportion 4-year-olds making a *backwards blocking* inference did not differ between this study and Sobel et al.’s first experiment, but did differ between this study and Sobel et al.’s second experiment. So overall, our data were highly comparable to previous data.

**Table 1:** Children’s responses on causal inference trials in the present study and comparison of these responses with previous studies; numbers (and percentages) of participants succeeding (S) and not succeeding (NS).

		Present study		Previous studies			Comparison
Trial type	Age in years	S	NS	S	NS	Authors (year, experiment)	Chi-square (df, *p-*value Fisher’s exact)
*Screening-off* (trial 1 present study)	2	13 (57)	10 (43)	18 (72)	7 (28)	S & K (2006, 2)	1.26 (1, .37)
	3	17 (74)	6 (26)				
	4	15 (65)	8 (35)				
	5	19 (91)	2 (9)				
*Indirect screening-off A* (trial 2 present study)	2	9 (39)	14 (61)				
	3	9 (39)	14 (61)				
	4	12 (52)	11 (38)				
	5	13 (62)	8 (38)				
*Indirect screening-off B* (trial 3 present study)	2	23 (100)	0 (0)				
	3	22 (96)	1 (4)	16 (100)	0 (0)	S et al. (2004, 1)	0.71 (1,1)
	4	22 (96)	1 (4)	16 (100)	0 (0)	S et al. (2004, 1)	0.71 (1,1)
				15 (94)	1 (6)	S et al. (2004, 2)	0.07 (1, 1)
	5	20 (95)	1 (5)				
*Backwards blocking* (trial 4 present study)	2	4 (17)	19 (83)				
	3	16 (70)	7 (30)	8 (50)	8 (50)	S et al. (2004, 1)	1.53 (1, .32)
	4	22 (96)	1 (4)	14 (88)	2 (13)	S et al. (2004, 1)	0.88 (1, .56)
				10 (63)	6 (38)	S et al. (2004, 2)	7.04 (1, .03*)
	5	21 (100)	0 (0)				

*Note*: S & K (2006) refers to Sobel and Kirkham (2006), S et al (2004) refers to Sobel et al., 2004. Please see Material and Methods section for the description of the trials and the specific responses on each trial consistent with making the intended causal inference, that is, succeeding. Note well: Sobel et al. (2004) administered both the indirect screening-off and the backwards blocking trials twice. They did not report the responses on the first and second administration separately, but did mention that these did not differ significantly. Therefore, in this Table we reported their results as if both administrations rendered exactly the same responses.

### Studying variability 1: Can variability in children’s behavior be explained by strategy use?

**Fig 1:**
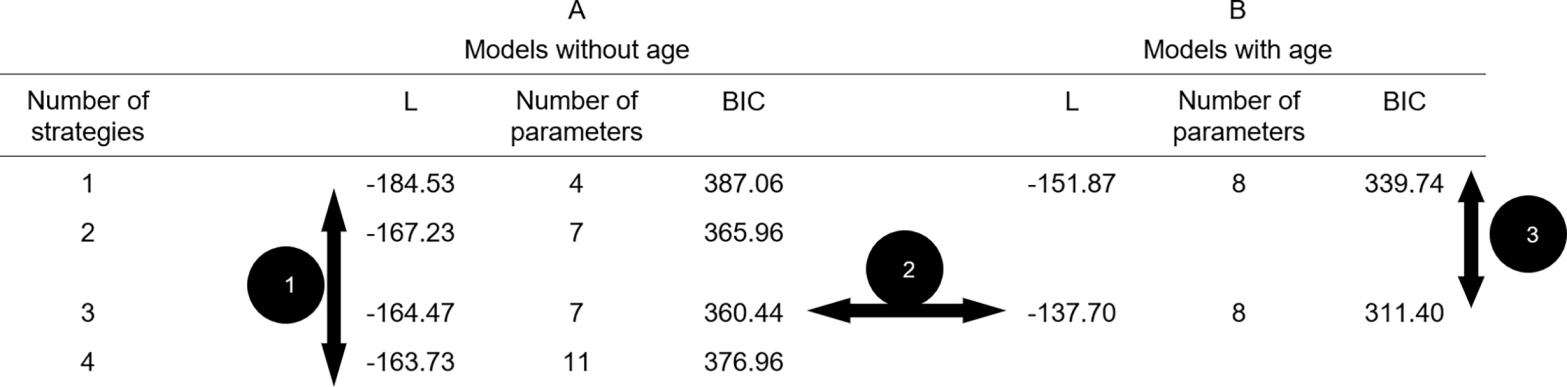
Goodness-of-fit measures for latent class models. *Note*: L = Log Likelihood, Number of parameters = number of feely estimated parameters minus the number of parameters estimated at the boundary, BIC = Bayesian Information Criterion. The arrows correspond to the first three steps in studying variability (see Results section).

First, we investigated whether the existence of different strategies could explain variability in children’s behavior (*strategy use*). To this end, latent class models (LCM; see Method) with 1, 2, 3, and 4 classes were fit to the data: children’s binary response patterns on four test trials. A 1-class-model showing the best fit, would indicate that variability could not explained by *strategy use*. A multiple-class-model showing the best fit, would indicate that variability could (at least partially) explained by *strategy use*. Fig 1A shows the goodness-of-fit measures of the 1-, 2-, 3-, and 4-class-models (see arrow 1). The 3-class-model had the lowest BIC value, implying the best and most parsimonious fit to the data. This demonstrates that variability in behavior was (at least partially) explained by *strategy use*.

### Studying variability 2: Is children’s strategy use related to age?

Next, we investigated whether children’s *strategy use* was related to age. To this end, a LCM with 3 classes and age in months as a covariate on the class membership probabilities (see Method) was fit to the data, and the fit of this model was compared to that of the original 3-class-model (see arrow 2 in Fig 1). Compared to the 3-class-model without age, the 3-class-model with age showed a better and more parsimonious fit to the data (likelihood ratio test: G2 = 53.54 with df = 2, *p* < .001). This demonstrates that *strategy use* was related to age.

### Studying variability 3: Does age-related strategy use better explain the variability in children’s behavior than age-related increase in accuracy of executing a single strategy?

Next, we investigated whether age-related *strategy use* better explained the variability in children’s behavior than age-related increase in *accuracy of executing* a single strategy. To this end, a LCM with 1 class and age in months as a covariate on the response probabilities (see Method) was fit to the data, and the fit of this model was compared to that of the 3-class-model with age (see arrow 3 in Fig 1). The 1-class-model with age showing a better fit, would indicate that children of different ages applied the same strategy, but became better at executing this strategy with age. The 3-class-model with age showing a better fit, would indicate that children of different ages applied different strategies. Fig 1B shows the goodness-of-fit measures of the 1-class-model with age. Compared to this model, the 3-class-model with age had a lower BIC value, implying a better and more parsimonious fit to the data. This demonstrates that age-related *strategy use* better explained the data than age-related increase in *accuracy of executing* a single strategy.

### Studying variability 4: The selected model

To conclude, on the basis of the above analyses a LCM with 3 classes and age as a covariate on class membership was selected. The selected model captures the data well as indicated by the likelihood ratio Chi-Square (G2 = 4.26 with df = 1). As there were a number of empty and small valued cells in the data table, the *p*-value for this G2 was obtained by using a parametric bootstrap procedure [32] and had value *p* = 0.35.

Table 2 shows the response probabilities for the selected model. These are the probabilities of succeeding on a specific trial given membership of a specific class. As mentioned above, succeeding was defined as making the intended causal inference, such as *screening-off* or *backwards blocking* (see Method). Children applying the first strategy had high probabilities of succeeding on all test trials. This was a relatively large group (N = 51) mostly consisting of children in the older age-range. Compared to children in the first group, children applying the second strategy had lower probabilities of succeeding on the first two test trials (*screening-off* and *indirect screening-off A)*. On the last two trials they showed a qualitatively different pattern of responses compared to first group: they succeeded on trial 3 (*indirect screening-off B*), but not on trial 4 (*backwards blocking*). This was a smaller group (N = 27) consisting of children in the younger age-range. Last, children applying the third strategy did not succeed on the first two trials (*screening-off* and *indirect screening-off A)*, but did succeed on the last two trials (*indirect screening-off B* and *backwards blocking)*. This was a relatively small group (N = 12) consisting of children of all ages.

**Table 2:** Selected latent class model: Response probabilities.

	Test trial 1 *screening-off*	Test trial 2 *indirect* *screening-off A*	Test trial 3 *indirect* *screening-off B*	Test trial 4 *backwards blocking*
Strategy 1	1	.67	.94	1
Strategy 2	.48	.33	1	0
Strategy 3	0	0	1	1

*Note*: Response probabilities are the probabilities of succeeding on a specific trial given membership of a specific class.

**Fig 2:**
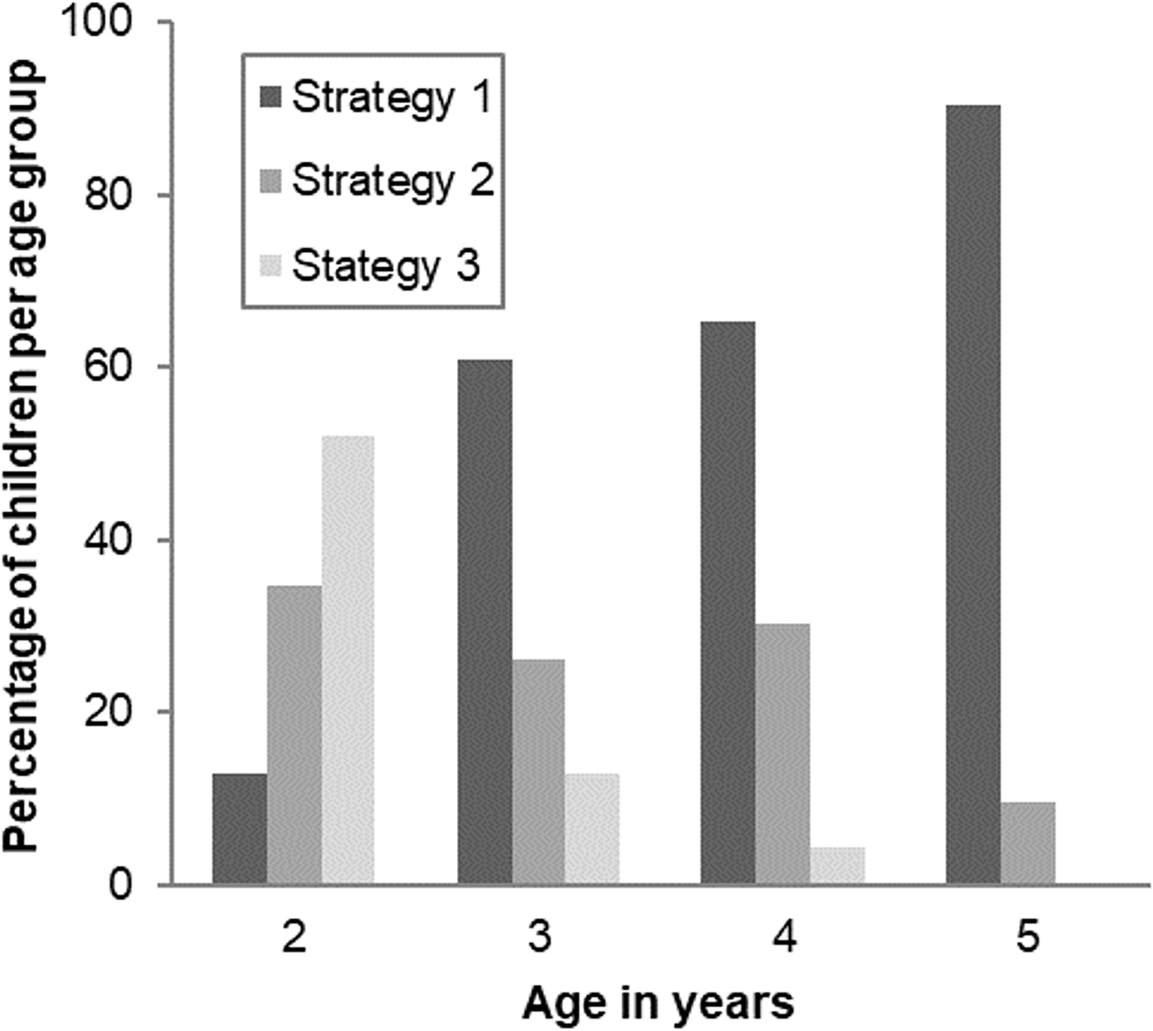
Selected latent class model: Percentages of children per strategy by age group.

Fig 2 shows how strategy use was related to age in the selected model (see Method). The use of the first strategy increased with age, and the use of the second strategy decreased with age. The majority of 2-year-olds applied the second strategy, while the majority of 3-, 4- and 5-year-olds applied the first strategy. In all age groups, a small proportion of children applied the third strategy.

## Discussion

The aim of the present study was to further investigate the development of causal inference by mapping the variability in young children’s behavior. Mainly, we set out to discriminate between two possible sources of variability, *strategy use* and *accuracy in strategy execution*, and relate these to age. In other words, we asked whether children of different ages apply different strategies when confronted with causal inference problems (*strategy use*), or whether they apply the same strategy, but become better at executing this strategy with age (*accuracy in strategy execution*). To the end of discriminating between these sources of variance, a relatively wide range of causal inference trials was administered to children of a relatively broad age range: 2- to 5-year-olds. Subsequently, individuals’ response patterns over trials were analyzed with a latent variable technique [13, 14]. It was shown that variability in behavior could largely be explained by age-related *strategy use*: children applied three different strategies when confronted with causal inference problems. And importantly, this age-related *strategy use* better explained the variability in children’s behavior than age-related increase in *accuracy of executing* a single strategy.

Hypotheses on development relating to both *strategy use* and *accuracy in strategy execution* have been proposed in the literature. Concerning *strategy use*, two types of strategies have been distinguished. First, children of different ages could apply different learning mechanisms. Both computational approaches, such as Bayesian methods [33, 34], and psychological approaches, such as causal learning on the basis of associative models [35, 36, 37] have been proposed (for an overview see Gopnik et al., 2004) [3]. Second, children of different ages could use different task-related strategies. For example, they could guess or imitate the experimenter, e.g. [5, 8]. Concerning *accuracy in strategy execution*, it has been suggested that with age children become better at executing a single strategy due to developing motor, statistical, or information processing abilities, or developing domain-specific knowledge [8, 9, 38]. For example, Sobel and Kirkham (2006) [8] demonstrated that in contrast to the majority of 24-month-olds, the majority of 19-month-olds failed to make *indirect screening-off* inferences in a blicket detector paradigm. The authors hypothesized this failure did not reflect the use of a different learning mechanism, but instead reflected the developing association between knowledge and action in the younger age group. By using an eye-gaze paradigm they found support for their hypothesis: 8-month-olds’ inferences were similar to those of the 24-month-olds. The present study’s results suggest a way of combining these existing hypotheses into a tentative model of the development of causal inference. It was shown that continuous development of motor, statistical or information processing abilities does not lead to a continuous increase in accuracy of executing a single strategy for causal inference. Instead, the development of causal inference is better described in terms of the application of multiple age-related strategies. This finding suggests that the development of certain abilities leads children to select and apply different strategies, either sequentially or according to an overlapping waves model [39, 40]. In other words, *strategy use* mediates the relation between children’s developing motor, statistical or information processing abilities and their causal inference. Previous work has found support for a mediatory role of *strategy use* in the development of transitive reasoning, free classification, and discrimination learning [22, 24, 25]. For example, Schmittmann et al. (2012) [24] found a relation between the use of discrimination learning strategies and children’s working memory and attentional control after controlling for age.

This study takes a first step in introducing a fruitful approach for investigating variability in behavior to the research on children’s causal inference. By using the approach we focused on the behavior that children of different ages demonstrate when confronted with causal inference problems, and the results can be considered indicative for children’s behavior when facing these types of problems in daily life. The findings show that 2- to 5-year-olds apply three different age-related strategies, that is, consistent patterns of behavior. Ultimately, one would want to know what learning mechanisms or task-induced strategies are reflected by these strategies, and how strategy use is related to age and the circumstances a child faces. Note that on the basis of this study’s results no definite conclusions can be drawn on the interpretations of the detected strategies. Future research, however, might address questions on the nature of strategies children of different ages apply by performing more confirmatory modeling approaches. Confirmative approaches require the formulation of specific hypotheses about the nature of the expected strategies. Therefore, in the following paragraph we elaborate on more and less plausible interpretations of the in the present study detected strategies based on the existing literature on children’s learning mechanisms and task-induced strategies. We do not suggest that, based on the data of the present study, definite conclusions can be drawn on interpretation of strategies, but with the following paragraph we intend to do suggestions for future work by elaborating on, in our opinion, more and less plausible interpretations.

Children applying the first strategy had high probabilities of succeeding on all test trials. The group’s responses are consistent with rational models, such as so called Bayesian inference over Causal Graphical Models, as proposed by Gopnik et al. (2004) [3] and Sobel et al. (2004) [9], but some authors claim that associative models could explain these data too [41]. One could possibly refer to this first strategy as the “rational strategy”. A clear difference between the first two groups was that children applying the second strategy showed a qualitatively different pattern of responses on the last two trials (*indirect screening-off B* and *backwards blocking*) compared to children applying the first strategy. As the associative strength of the target object is the same in these trials (that is, in both trials the target object has a 100% positive association with the outcome), the symmetry in children’s classification of the target object as a blicket on both these trials suggests the possible use of a learning mechanism on the basis of an associative model, such as the Rescorla-Wagner model [9, 37]. This interpretation is consistent with the group’s lower age and the fact that associative reasoning is a powerful learning mechanism early in life [42, 43]. Note that the pattern of responses does not reflect a yes bias, as the large majority of this group (89%) did not label the non-target object as a blicket on trial 3 (*indirect screening-off B)*. However, this hypothesis is inconsistent with the results if the rational model would suggest continuous development. One could possibly refer to this second strategy as the “associative strategy”. Children applying the third strategy did not succeed on the first two trials (*screening-off* and *indirect screening-off A*), but did succeed on the last two trials (*indirect screening-off B* and *backwards blocking*). A closer look at this group’s responses on the first two trials shows that the majority (70% on trial 1 and 61% on trial 2) responded by putting the causally effective block plus one or two ineffective blocks on the machine. These responses suggest the possible use of a task-related strategy directed at avoiding uncertainty: children in this group might have chosen to respond in a manner that they had observed to work most frequently or simply by putting all available objects on the machine. One could possibly refer to this third strategy as the “uncertainty avoidance strategy”. Looking at the developmental pattern, it was shown that the use of the “rational strategy” increased with age, the use of the “associative strategy” decreased with age, and the “uncertainty avoidance strategy” was applied by a small proportion of children in all age groups. Hence, there are appealing developmental interpretations of the model outcomes, but more research is needed to be able to draw more definite conclusions about the reasoning processes leading to the observed, different types of response patterns.

Several recommendations could be given for future work. To be able to interpret detected strategies in more detail, the mostly exploratory latent variable approach of the present study could be replaced by a more confirmatory approach. Using Rule Assessment Methodology [26, 44], trials could be selected that optimally distinguish between children applying different learning mechanisms [26, 44]. For example, base rate trials could be included to distinguish response patterns consistent with Bayesian inference over Causal Graphical Models from response patterns consistent with certain associative models [9, 45, 46]. Or, alternatively children's inference models could be estimated directly from data by adopting one causal reasoning framework, cf. [47]. In addition, the variability approach could be used to investigate whether strategy use differs over domains, such as the physical and psychological domains [7, 46, 48], or over task characteristics, such as deterministic or probabilistic tasks [49, 50, 51], and how strategy use is related to age under these different circumstances. Moreover, it is of high interest to relate children’s cognitive abilities, such as their statistical or information processing abilities, to their causal inference strategy, cf. [24]. To conclude, the approach for investigating variability in behavior has shed a new light on the development causal inference, and holds the promise of initiating a new line of research.
